# The Potential Role of Pharmacogenomic and Genomic in the Adjuvant Treatment of Early Stage Non Small Cell Lung Cancer

**DOI:** 10.2174/138920208784533665

**Published:** 2008-06

**Authors:** Clorinda Schettino, Maria A Bareschino, Paolo Maione, Antonio Rossi, Fortunato Ciardiello, Cesare Gridelli

**Affiliations:** 1 Division of Medical Oncology, Department of Clinical and Experimental Medicine and Surgery ‘F. Magrassi and A. Lanzara’, Second University of Naples, School of Medicine Via S. Pansini 5, 80131 Naples, Italy; 2 Division of Medical Oncology, “S.G. Moscati” Hospital, Avellino, Italy

**Keywords:** NSCLC, adjuvant treatment, molecular markers, ERCC1, RRM1, β-tubulin, EGFR.

## Abstract

Although notable progress has been made in the treatment of non-small-cell lung cancer (NSCLC) in recent years, this disease is still associated with a poor prognosis. Despite early-stage NSCLC is considered a potentially curable disease following complete resection, the majority of patients relapse and eventually die after surgery. Adjuvant chemotherapy prolongs survival, altough the absolute improvement in 5-year overall survival is only approximately 5%.

Trying to understand the role of genes which could affect drug activity and response to treatment is a major challenge for establishing an individualised chemotherapy according to the specific genetic profile of each patient. Among genes involved in the DNA repair system, the excision repair cross-complementing 1 (ERCC1) is a useful markers of clinical resistance to platinum-based chemotherapy. In the International Lung Cancer Trial (IALT) adjuvant chemotherapy significantly prolonged survival among patients with ERCC1 negative tumors but not among ERCC1-positive patients. BRCA1 and ribonucleotide reductase M1 (RRM1), two other key enzymes in DNA synthesis and repair, appear to be modulators of drug sensitivity and may provide additional information for customizing adjuvant chemotherapy.

Several clinical trials suggest that overexpression of class III β-tubulin is an adverse prognostic factor in cancer since it could be responsible for resistance to anti-tubulin agents. A retrospective analysis of NCIC JBR.10 trial showed that high tubulin III expression is associated with a higher risk of relapse following surgery alone but also with a higher probability of benefit from adjuvant cisplatin plus vinorelbine chemotherapy.

Finally, the use of gene expression patterns such as the lung metagene model could provide a potential mechanism to refine the estimation of a patient’s risk of disease recurrence and could affect treatment decision in the management of early stage of NSCLC.

In this review we will discuss the potential role of pharmacogenomic approaches to guide the medical treatment of early stage NSCLC.

## INTRODUCTION

Worldwide lung cancer accounts for the largest number of new cases of cancer and results in about 1 million of deaths/year [1]. For early-stage non small cell lung cancer (NSCLC) surgical lung resection is the treatment of choice; however, five-years survival rates range from 30% to 60% [2].

The role of adjuvant chemotherapy for resected early stage NSCLC was still an open question until few years ago, but currently it represents the standard treatment for patients with resected stage II-III disease. The results of the meta-analysis by the British Medical Research Council have demonstrated that cisplatin-based adjuvant chemotherapy for stage I through III NSCLC causes a non statistically significant advantage in survival of 5% at five years [3]. The first phase III clinical trials conducted after the meta-analysis have failed to demonstrate a significant benefit with adjuvant chemotherapy [4-6]. However, adjuvant chemotherapy has recently been re-evaluated in several clinical trials [7-12]. The International Adjuvant Lung Trial (IALT) reported a statistically significant 4% survival advantage at 5 years [hazard ratio (HR): 0.86; P< 0.03] with the addition of four cycles of cisplatin-based chemotherapy after complete resection of stage I–III NSCLC. The study was not stratified to evaluate results by stage, but a trend toward a greater benefit in stage II and III patients was identified [7]. In the JBR.10 trial patients with completely resected stage IB–IIB NSCLC were randomized to receive four cycles of chemotherapy (cisplatin/ vinorelbine) or only observation [9]. A 15% survival advantage was reported at 5 years (HR:0.70; P=0.012) in the chemotherapy arm. The trial was stratified by stage, and, in a subset analysis, no benefit was noted for stage IB patients (HR:0.94). In the only adjuvant trial to use a carboplatin-based regimen (CALGB 9633) in patients with resected stage IB NSCLC receiving adjuvant chemotherapy a survival advantage was initially reported. This trial was closed early when the first interim analysis demonstrated a 12% survival advantage at 4 years (HR:0.62) [10]. However, an update of the CALGB 9633 study, which was presented at the American Society of Clinical Oncology (ASCO) 2006 Annual Meeting showed no significant survival advantage for adjuvant chemotherapy [11]. Confirmation of beneficial role of adjuvant therapy was also demonstrated in the Adjuvant Navelbine International Trialist Association (ANITA) trial. In this study, patients with resected NSCLC had a 9% survival advantage at 5 years (HR:0.79; P=0.013) with adjuvant chemotherapy (cisplatin/vinorelbine), but no benefit was found for stage IB NSCLC patients [HR:1.10; 95% confidence interval (CI), 0.76–1.57] [12].

An individual patient meta-analysis (LACE) of the five largest cisplatin-based studies (ALPI, BLT, IALT, JBR.10, and ANITA) has been conducted. This study found a 5.3% absolute survival advantage at 5 years (HR:0.89; 95% CI, 0.82–0.96; P=0.004) for adjuvant cisplatin therapy. However, this benefit depends on stage and is greatest in patients with stages II and III (HR:0.83; 95% CI, 0.73–0.95) while a detriment for chemotherapy was suggested in stage IA patients (HR:1.41; 95% CI, 0.96–2.09) [13]. 

Therefore, adjuvant cisplatin-based chemotherapy is currently recommended for fully resected stage II and III NSCLC [14]. 

Moreover, the majority of patients, which are enrolled in clinical trials of adjuvant chemotherapy are patients selected for relative better prognostic factors, such as younger age, good performance status (PS) and clinically fit to receive platinum-based chemotherapy. Therefore, these results are not directly translatable to daily clinical practice. Further, there are no data regarding the role of adjuvant chemotherapy in patients with pure bronchioloalveolar histology carcinoma, in elderly patients (age> 75 years) and in patients receiving adjuvant treatment after two months from surgery. Sex, histologic type, age and other clinical variables are validated prognostic factor but do not allow to select those patients who could benefit from chemotherapy [7,13]. Stage is still the most important factor to consider when administering adjuvant treatment. However, there is a need for new approaches including the use of molecular markers for selecting patients who are most likely to benefit from a given adjuvant therapy. 

## MOLECULAR PROGNOSTIC AND PREDICTIVE FACTORS 

Recently, an increasing body of experimental evidence has been provided to link tumor-specific molecular characteristics, such as epidermal growth factor receptor (EGFR) mutations and/or EGFR gene copy number with response to the EGFR inhibitors gefitinib and erlotinib in metastatic NSCLC [15-17]. Likewise, current data suggest that several molecular markers may be predictive of response to specific cytotoxic drugs in certain subpopulation of NSCLC patients.

### Excision Repair Cross-Complementation Group 1 (ERCC-1) and Ribonucleotide Reductase Subunit M1 (RRM1)

#### ERCC1

Platinum compounds exert their cytotoxic effects by binding covalently to genomic DNA, forming adducts that result in altered forms of DNA thus interfering with normal cellular replication. To maintain the structural integrity of DNA, an intricate network of DNA repair systems is involved. One of the most important is the nucleotide excision repair (NER), a highly versatile and sophisticated DNA damage removal pathway. ERCC-1 is component of the NER complex, and could be measured to assess the functional status of this pathway. It forms a molecular complex with another protein, belonging to the xeroderma pigmentosum group F (XPF), and removes modified nucleotides together with adjacent nucleotides from the damaged strand during the first step (excision), which is followed by synthesis of an intact strand through DNA polymerase activity (repair synthesis) [18]. *In vitro* studies have linked platinum resistance to the expression of ERCC1 messenger RNA (mRNA) in ovarian, cervical, testicular, bladder and NSCLC cancer cell lines [19]. The relationship between the expression of ERCC1 mRNA and platinum resistance has been observed in small, retrospective clinical trials in patients with several malignancies such advanced gastric, ovarian, colorectal, esophageal, or NSCLC [20-23]. Furthermore, two common polymorphisms of the ERCC1 gene [24-27] (codon 118 C/T and C8092A) have been correlated with the response to platinum-based chemotherapy in colorectal cancer [28] and NSCLC [29]. These polymorphisms are mainly associated with lower rates of translation of the ERCC1 gene, which results in low levels of the protein into the nuclei.

The influence of ERCC-1 on resistance to chemotherapy in the adjuvant setting of NSCLC patients has been evaluated in a retrospective analysis of the IALT trial. Seven hundred and sixty one tumor specimens were analyzed to evaluate the expression of ERCC-1 protein by immunohistochemistry (IHC). Among these, 426 (56%) samples were scored as negative, while 335 (44%) samples were ERCC1-positive. A Multivariate logistic model showed that the expression of ERCC1 was significantly correlated with age (P=0.03; less common in patients younger than 55 years of age than in patients 55 to 64 years of age), histologic type (P<0.001; less common in adenocarcinomas than in squamous-cell carcinomas), and pleural invasion (P=0.01; less common in the absence than in the presence of pleural invasion).

Among patients with ERCC1-negative tumors, overall survival (OS) was significantly longer in the chemotherapy group compared to surgery alone (HR:0.65; 95% CI, 0.50 to 0.86; P=0.002). Median survival time (MST) resulted 14 months longer in patients receiving adjuvant treatment (56 months) than in the control group (42 months). Also disease free survival (DFS) in ERCC1-negative patients was longer in the chemotherapy group than in the control group (HR: 0.65; 95% CI, 0.50 to 0.85; P=0.001). ERCC1-positive patients did not experience any benefit from adjuvant cisplatin based chemotherapy compared with ERCC1-positive patients in the control group (HR:1.14; 95% CI, 0.84 to 1.55; P=0.40). Analyzing exclusively patients in the control group, the 5-year overall survival (OS) rate was significantly higher (46%) among patients with ERCC1-positive tumors as compared to ERCC1-negative patients (39%) (HR:0.66 95% CI, 0.49 to 0.90; P=0.009) [30]. These results led to the hypothesis that determination of ERCC1 expression in fully resected NSCLC can be an independent predictor of the efficacy of adjuvant chemotherapy. 

With the increasing evidence supporting ERCC-1 expression as a marker of platinum resistance and worse prognosis in patients with metastatic NSCLC [31-32], several research groups are planning prospective clinical trials using ERCC1 to guide treatment decisions.

The first prospective trial testing the concept of customized chemotherapy in NSCLC is a phase III study in which four hundred and forty four metastatic NSCLC patients were randomly assigned in a 1:2 ratio to either the control arm or the genotypic arm. According to ERCC1 mRNA baseline levels, patients received cisplatin plus docetaxel (low ERCC1 mRNA), or a non platinum-based regimen, such as gemcitabine plus docetaxel (high ERCC1 mRNA), while all patients in the control arm received cisplatin plus docetaxel. Among 346 patients assessable for response (primary endpoint), 53 patients (39.3%) in the control arm and 107 patients (51.2%) in the genotypic arm achieved objective response (one-sided Fisher's exact test, P=0.02). In an exploratory analysis of response, the genotypic arm was subdivided into the low and high ERCC-1 mRNA level groups. Sixty-five patients (53.2%) in the low genotypic group, and 42 patients (47.2%); in the high genotypic group achieved objective responses [33]. There was no difference in survival rates between the two treatment strategies (9.8 months in the control arm vs 9.9 months in genotypic arm P=0.59), although the study was not powered to assess survival as its primary end point [33]. 

#### RRM1

Another key enzyme in DNA synthesis and repair is the ribonucleotide reductase. It is the only known enzyme that converts ribonucleotides into deoxyribonucleotides which are required for DNA synthesis and repair. The ribonucleotide reductase consists of two dimerized subunits (RRM1 and RRM2), whose dimerization is required for DNA synthesis [34].

RRM1 gene expression has been identified as a potential pharmacogenetic marker of gemcitabine resistance. Gemcitabine (2’, 2’-difluorodeoxycitidine) is one of the most widely used cytotoxic drugs for NSCLC treatment, which interfere with ribonucleotide reductase [35,36]. The overexpression of RRM1 has been linked to the tumor resistance to platinum drugs and gemcitabine [32] while high expression of RRM1 is a positive prognostic marker for survival probably because its induces phosphatase and tensin homolog (PTEN) expression and inhibit cell migration, invasion, and metastasis formation [37,38].

Retrospective data from patients with stage IV NSCLC have provided evidence that RRM1 mRNA expression is a key predictive marker of survival in patients treated with gemcitabine plus cisplatin [39,40].

The correlation between ERCC1, RRM1 mRNA levels and shortened survival has been observed by Ceppi *et al. *in advanced NSCLC patients receiving platinum- and gemcitabine-based chemotherapy [32]. Recently, Zheng *et al. *have analyzed RRM1, ERCC1 and PTEN protein expression with an automated quantitative determination, in histological specimens from early stage (stage I) NSCLC patients treated with surgery alone. RRM1 expression correlated with the expression of ERCC1 (P< 0.001), but not with the expression of PTEN (P=0.37). The DFS in patients with RRM1 high levels was more than 120 months compared to the DFS of 54.5 months observed in patients with low RRM1 levels; this difference was statistically significant (P=0.004 HR for low vs high expression, 2.2). The median OS was 120 months and 60 months, respectively (HR:0.60; P=0.02). In this trial the expression of RRM1 was not associated with tumour stage, histologic type, age, sex, Eastern Cooperative Oncology Group (ECOG) PS, absence or presence of weight loss and smoking status. The investigators confirmed that RRM1 expression correlate with the expression of ERCC1 (P=0.001). One hundred and eighty four patients whose tumor specimens were scored for both proteins were separated in four group. Only patients who had high expression of both proteins had a median DFS and median OS of more than 120 months, that were significantly longer than those for patients in the other groups (P=0.01 for DFS, P=0.02 for the OS). These data suggest that the concomitant high expression of both proteins delineate a subgroup of patients (approximately 30%) with a relatively better outcome [41]. 

A prospective, phase II clinical trial that used tumor expression of RRM1 and ERCC1 mRNA as detected by real-time quantitative polymerase chain reaction (RT-QPCR) for selection of chemotherapy in advanced NSCLC patients has been recently conducted. This strategy resulted in four possible gene expression strata with the following therapies: the low RRM1 and low ERCC1 group (gemcitabine and carboplatin treatment group); low RRM1 and high ERCC1 group (gemcitabine and docetaxel treatment group); the high RRM1 and low ERCC1 group (docetaxel and carboplatin treatment group); the high RRM1 and high ERCC1 group (docetaxel and vinorelbine treatment group). Among eighty five patients enrolled, sixty underwent tumor biopsy and gene expression analysis was performed in fifty five patients. Response Rate (RR) was 44%, OS was 59% and PFS 14% at 1 years, with a median of 13.3 and 6.6 months, respectively [42]. Considering these data, a prospective phase II clinical trial to assess the efficacy of selecting chemotherapy regimens (with or without platinum, and with or without gemcitabine) based on ERCC1 and RRM1 tumor expression in adjuvant setting of NSCLC has been planned by the Southwest Oncology Group (SWOG). 

#### p27 ^kip1^

Biomarkers that are involved in cell cycle regulation have become of interest as potential predictors of outcome in adjuvant chemotherapy. Cyclins, their associated cyclin-dependent kinases, and cyclin-dependent kinase inhibitory proteins play a central role in cell cycle progression and may also affect response to chemotherapy [43,44]. *In vitro* studies suggested that overexpression of p27^Kip1^ is associated with resistance to anticancer drugs [44-46]. Up regulation of p27^Kip1^ mediates drug resistance both in tumor cells and protects small-cell lung carcinomas from apoptosis, whereas downregulation of p27^Kip1 ^expression by antisense oligonucleotides reduces intercellular adhesion, increases cell proliferation, and enhances drug sensitivity of tumor cells [44-47]. Therefore, p27^Kip1^ downregulation may contribute to chemotherapy sensitivity by cell cycle transition delay and by increased susceptibility to apoptosis. The potential prognostic and/or predictive value of cell cycle regulators (p27^Kip1^, p16^INK4A^, cyclin D1, cyclin D3, cyclin E, and Ki-67) has been evaluated in NSCLC patients who were enrolled in the IALT trial. These biomarkers were assessed by IHC analysis in tumor sections from 783 patients. A relationship has been observed between the p27^Kip1^ status and the benefit of cisplatin-based chemotherapy (P=0.02). Among patients with p27^Kip1^-negative tumors, patients who were treated with cisplatin-based chemotherapy had a longer OS time than patients in the control group (HR:0.66; 95% CI, 0.50 to 0.88; P=0.006). In patients with p27^Kip1^-positive tumors, the OS was not different between patients treated with chemotherapy and controls (HR:1.09; 95% CI, 0.82 to 1.45; P=0.54). Furthermore, in p27^Kip1^-negative patients, the MST was 13 months longer in the chemotherapy group compared with the control group (58 and 45 months, respectively). Comparable results were obtained when the p27^Kip1^ score was analyzed as a continuous variable (P=0.03). When the analysis was focused on the chemotherapy arm, patients with p27^Kip1^-positive tumors experienced a shorter OS compared with patients with p27^Kip1^-negative tumors (HR:1.35; 95% CI, 1.02 to 1.80; P=0.04). In contrast to p27^Kip1^, none of other cell cycle regulators was able to predict the benefit for adjuvant chemotherapy in this study [48].

Since both ERCC1 and p27^Kip1 ^could be independent predictive factors in patients with resected NSCLC receiving adjuvant cisplatin-based chemotherapy, combining the determination of the expression of these proteins their predictive value could further increase. In the molecular analysis of the IALT trial, the major benefit from adjuvant treatment was observed in patients whose tumor specimens were negative for both biomarkers (HR 0.52, 95% CI 0.36-0.74) as compared to patients whose cancer were positive for both proteins (HR:1.27, 95% CI 0.77-1.84) [49].

#### Class III β-Tubulin 

β-Tubulin is one of the major components of microtubules which are complex polymers consisting of tubulin dimers (containing one α tubulin plus one β tubulin molecule) and a variety of mictotubule-associated proteins (MAPs), including τ protein, microtubule associated protein 4, and stable tubule-only polypeptide proteins [50].

In humans, at least six distinct β-tubulin isotypes (classes I, II, III, Iva, IVb, and V) have been reported, and their expression profile differs among tissues [51]. Class III β-tubulin (βTubIII) differs from other tubulin isotypes in its amino acid sequence and post-translational modifications, which include phosphorylation and polyglutamylation [52]. *In vitro* studies showed that up-regulation of βTubIII in lung and ovarian tumor cell lines confered resistance to docetaxel/paclitaxel [53–55]. Furthermore, overexpression of βTubIII in advanced ovarian, breast, and gastric cancers is generally associated with resistance to paclitaxel treatment and with a poor prognosis [56–58]. Several studies have shown that the level of βTubIII (assessed by IHC or other techniques) may be both a prognostic and a predictive factor in advanced NSCLC. Twenty-five tumor specimens from advanced NSCLC patients treated with vinorelbine/cisplatin have been analyzed using QPCR. High βTubIII mRNA levels correlated with inferior outcome in advanced NSCLC patients treated with anti-tubulin agents [39]. It has also been shown a relationship between the high level of expression of βTubIII in NSCLC tumor samples of patients treated with vinorelbine and the rate of progression following therapy. High expression of βTubIII also correlated with shorter PFS and OS (P=0.002 and P=0.001 respectively) [59].

In advanced NSCLC patients treated with paclitaxel-based regimens, whose tumors expressed low levels of class III β tubulin isotype, a better RR, longer PFS and OS (P< 0.001, 0.004, and 0.002, respectively) have been reported [60]. The effect of βTubIII on patient outcome and benefit from adjuvant chemotherapy has been recently investigated. Tumor tissues from 265 of the 482 patients enrolled in the NCIC-JBR.10 trial were analyzed for βTubIII expression by IHC. High tubulin expression was significantly associated with poorer relapse-free survival (RFS) (HR:1.92; 95% CI, 1.16-3.18; P=0.01), and a similar trend was seen for OS (HR:1.72; 95% CI, 1.02-2.88; P=0.04) in absence of adjuvant chemotherapy treatment; conversely tubulin expression was not a statistically significant predictor of outcome in the patients assigned to receive chemotherapy: RFS (HR:1.10; 95% CI, 0.62-1.95; P=0.75), OS (HR:1.11; 95% CI, 0.65-1.88; P=0.7). The adverse prognostic value of high tubulin III expression is in accord to prior data reported in advanced NSCLC setting. Among the 133 high tubulin expressor patients, those receiving chemotherapy (68/133) had significantly improved RFS (HR:0.45; 95% CI, 0.27-0.75; P= 0.002) and a trend toward improved OS (HR:0.64; 95% CI, 0.39-1.04; P=0.07) as compared with patients (65/133) in the observation arm. In the low tubulin group, no significant difference in RFS (HR:0.78; 95% CI, 0.44-1.37; P=0.4) or OS (HR:1.00; 95% CI, 0.57-1.75; P=0.99) was seen according to treatment assignment (surgery alone or adjuvant chemotherapy) [61]. These results are in contrast to the data observed in advanced NSCLC, in which low tubulin III expression was correlated to higher RR to chemotherapy regimens which contain anti-microtubule drugs [39,59-60]. However, considering these results, further evaluation of the role of βTubIII as a marker of chemosensitivity is necessary.

#### Kirsten Rat Sarcoma Viral Oncogene Homolog (K-Ras) 

The small guanosine triphosphate (GTP) binding protein Ras plays an important role in mediating multiple intracellular signal transduction pathways, including growth, apoptosis, and differentiation. Active GTP-bound Ras interacts with its downstream effectors and regulates the transduction of proliferative signals through the Raf/mitogen-activated protein kinase pathway and of the apoptosis through the Nore1/*Mst*I and phosphatidylinositol 3'-kinase pathway [62].

Activating mutations in the RAS family member were found approximately in 15-30% of lung cancers and resulted in MAPK pathway activation. These mutations are most frequently detected in codons 12 and 13 in the exon 2 of the K-RAS gene [63,64] and in patients with a history of substantial cigarette use [65].

Among 482 patients with completely resected early stage NSCLC enrolled in the JBR-10 clinical trial, the status of ras mutation (analysis of codons 12,13 and 21 of Harvey-ras, K-ras and N-ras) was evaluated in 450 (93%) tumor specimens. K-ras mutations were found in 24% of patients. In the patients with wild type K-ras gene who did not receive chemotherapy the median survival was 74 months, whereas it was not reached in chemotherapy group (HR:0.69, 95% CI 0.49-0.98, P=0.03). In patients whose tumors contained K-ras mutations, adjuvant chemotherapy conversely did not improve survival (HR:0.95, 95% CI 0.53-1.71, P=0.87) [9].

#### EGFR 

The Epidermal Growth Factor Receptor (EGFR) is a member of the ErbB family of cell membrane growth factor receptors that are important mediators of cell growth, differentiation, and survival. EGFR is a 170 KDa transmembrane glycoprotein, with an intracellular domain that serves as the site of protein tyrosine kinase activity [66]. The EGFR is frequently overexpressed and/or abnormally activated in 40-80 % of NSCLC [67]. The blockade of EGFR signalling in cancer cells not only determines the inhibition of cell proliferation but also has other effects that could be relevant for the clinical activity including induction of apoptosis; anti-angiogenesis through inhibition of angiogenic growth factor production; inhibition of invasion and metastasis; potentiation of anti-tumor activity of cytotoxic drugs and of radiotherapy [68].

Two types of anti-EGFR targeting agents have reached advanced clinical development: monoclonal antibodies (Mabs) and small molecule inhibitors of the EGFR tyrosine kinase enzymatic activity (TKIs). Among EGFR-TKIs, erlotinib is currently approved worldwide for the treatment of chemotherapy-resistant advanced NSCLC patients, while the use of gefitinib is restricted to Asian countries.

Several researchers have identified somatic mutations in the EGFR gene and have discovered that these mutations were associated with a higher likelihood of clinical response to treatment with gefitinib and erlotinib [15-16,69]. EGFR gene mutations were most frequently detected in a subpopulation of NSCLC patients with characteristics associated with a better treatment outcome: female sex, non smokers patients, Asian origin, adenocarcinoma histology. The more common EGFR mutations are an in frame deletion in exon 19 around codons 746 to 750 and a missense mutation leading to leucine to arginine substitution at codon 858 (L858R) in exon 21 [70]. In a large number of retrospective clinical trials, it has been suggested that NSCLC patients treated with EGFR-TKIs, whose tumors are carrying sensitizing EGFR mutations achieve an improvement of survival [71-78], although this effect has not always been demonstrated [17,79-81]. 

Different somatic EGFR gene mutations may confer diverse tumor activation profiles that lead to variations in both natural history and clinical course after treatment with erlotinib or gefitinib. The relationship between the two most common types of somatic EGFR mutations has been evaluated. Emerging data suggest that patients with NSCLC and EGFR exon 19 deletion have a longer survival following treatment with gefitinib or erlotinib compared with those with the L858R mutation [82-84].

Several prospective clinical trials have been conducted to determine the RR to EGFR-TKIs in Caucasian and in East Asian patients carrying EGFR somantic mutations [85-89]; these data suggest that EGFR-TKIs are highly active treatment for patients harbouring EGFR gene mutations; however, at this time there are no data from prospective randomized clinical trial which demonstrate a survival advantage for NSCLC patients with EGFR mutation treated with EGFR-TKIs.

The potential predictive role of EGFR gene amplification by fluorescence *in situ* hybridization (FISH) in patients treated with gefitinib or erlotinib is emerging from several retrospective analyses [17,76]. In the retrospective analysis of a phase III randomized, double-blind, placebo-controlled trial (the BR21 study), EGFR FISH-positive patients randomized to receive erlotinib had a significantly superior survival (HR:0.44, P=0.01), as compared with FISH-positive patients randomized into the placebo arm (HR:0.44, P=0.01). Similar results were observed in the Iressa Survival Evaluation in Lung cancer (ISEL) study, a randomized, placebo-controlled, phase III clinical trial, that has investigated the effect of gefitinib on survival in chemotherapy refractory advanced NSCLC patients. Significantly, EGFR FISH-positive status was associated with certain clinical and biological characteristics predictive for EGFR-TKI sensitivity, such as female sex, never-smoking history, and the presence of EGFR mutations. In another phase III clinical trial, the Iressa NSCLC Trial Evaluating Response and Survival Against Taxotere (INTEREST trial), which has met the primary endpoint of noninferiority of gefitinib as compared to docetaxel, patients with high EGFR gene copy numbers receiving gefitinib had a MST of 8.4 months as compared to 7.5 months in those treated with chemotherapy (HR:1.09; 95 %CI 0.78-1.15 P=0.6199) [90]. In a phase II randomized study named IRESSA in NSCLC vs Vinorelbine Investigation in The Elderly (INVITE trial), comparing gefitinib to vinorelbine in chemonaïve elderly patients with advanced NSCLC, PFS, the primary end point of this study, was not significantly different between the two arms (2.7 and 2.9 months for gefitinib and navelbine, respectively). Exploratory analyses in EGFR FISH-positive subgroup, HRs for gefitinib as compared to vinorelbine were 3.13 (95% CI 1.45, 6.76) for PFS and 2.88 (95% CI 1.21, 6.83) for OS, surprisingly favoring chemotherapy to gefitnib treatment [91].

The data emerging from advanced stage NSCLC patient warranted the investigation of EGFR inhibitors in the adjuvant setting. However, two adjuvant trials of gefitinib vs placebo in unselected patients have been stopped early in Japan, due to toxicity (interstitial lung disease), and in Canada, due to negative results of the ISEL trial in advanced disease. To date, erlotinib is under investigation in adjuvant setting in selected patients. In the Randomized phase III double-blind trial in adjuvant NSCLC with Tarceva (RADIANT trial) patients with resected stage IB to IIIA NSCLC which are positive for EGFR detected by FISH or IHC are being enrolled. Patients are randomized to receive two years of erlotinib at standard dose versus placebo after four cycles of adjuvant chemotherapy containing platinum agents. In this study, it will be also retrospectively analyzed the mutational status of EGFR and K-ras genes. 

Nevertheless, it may be of major interesting to plan clinical trials in the adjuvant setting by selecting patients through the knowledge of EGFR or K-ras gene mutations.

### Other Potential Biological Markers

#### Multidrug Resistance Proteins (MRP)

The MRP family, also known as C group of adenosine triphosphate (ATP)-binding cassette transporters, consists of 13 structurally related ATP-binding cassette transporters that are involved in the transport of various molecules. Overexpression of MRP1 or MRP2 in tumor cells confers resistance to various anticancer drugs [92-93].

The prognostic and/or predictive value of the different MRPs in malignant diseases is under investigation. Recently, IHC analysis of MRP1 and MRP2 was retrospectively performed in tumor sections of 782 patients from IALT trial. Patients with MRP2-positive tumors had a significantly shorter OS than patients with MRP2-negative tumors [HR:1.37; 95% CI, 1.09-1.72; P=0.007]. Median OS was 9 months longer in the MRP2-negative group compared with MRP2-positive tumors (54 and 45 months, respectively). In contrast, no significant correlation between MRP1 expression and OS of the patients was reported. 

However neither the expression of MRP1 nor of MRP2 is associated to the benefit of cisplatin-based chemotherapy [94]. 

#### Apoptotic Markers

An analysis of apoptotic markers such as the death receptor Fas, its ligand FasL and survivin, a G2-M cell cycle regulated protein with anti-apoptotic properties, has been recently performed. Among 773 specimens from the IALT trial which were analyzed by IHC, 73% were Fas negative, 49% FasL negative and 54% survivin positive. Fas positivity correlated with vascular invasion, while FasL correlated with histology (higher in adenocarcinoma) and pleural invasion. Survivin was lower in adenocarcinoma, higher in males and lower in older patients. None of these biomarkers appears to be related to prognosis; only the ratio of a Fas to FasL > or = 1 was associated with longer survival (HR:0.72; P=0.02). None of these markers had predictive value for chemosensitivity at the level of significance 0.01. However, a borderline predictive value was observed for FasL negative (HR:0.69) as compared with FasL positive (HR:1.03; P=0.06) samples, as well as for Fas:FasL ratio >1 (HR:0.51) as compared with a ratio of 1 (HR:1.13) or a ratio ≤1 (HR:0.80; P=0.05) [95].

#### BRCA1

BRCA1 is implicated in transcription-coupled nucleotide excision repair (TC-NER), and modulation of its expression determine modification of TC-NER and then radio and chemo-resistance. It is a component of a large DNA repair complex named the BRCA1-associated genome surveillance complex, containing mismatch repair proteins. Therefore, BRCA1 may be a potential role in mismatch repair mechanisms [96-97]. BRCA1 overexpression has been correlated to apoptosis through the c-Jun N-terminal kinase pathway [98], which is activated by cisplatin-induced DNA damage [99].

*In vitro* studies have shown that BRCA1 can regulate differential sensitivity to different classes of chemotherapy agents [100]. It has been demonstrated that BRCA1 abrogates the apoptotic phenotype induced by a range of cytotoxic agents such as cisplatin, bleomicin, and determines remarkable response to several antimicrotubule drugs [100-102]. On the basis of the evidence for the role of BRCA1 in breast and ovarian cancers, it has been suggested that BRCA1 mRNA expression could also play an important role in predicting differential chemotherapy sensitivity in NSCLC. BRCA1 mRNA expression has been evaluated in 55 surgically resected NSCLC patients who had received neoadjuvant gemcitabine/cisplatin chemotherapy by RT-QPCR. Patients were divided according to the gene expression values into quartiles. In these locally advanced NSCLC patients, BRCA1 mRNA expression seems to predict outcome. Median survival was not reached for patients with low BRCA1 level, whereas in the two middle quartiles, it was 37.8 months (95% CI, 10.6–65), and for the 12 patients in the top quartile, it was 12.7 months (95% CI, 0.28-28.8) (P=0.01) [103]. This findings may be indicative that patients with high levels could better respond to non platinum-based chemotherapy but more to antimicrotubules agents.

A pilot study of adjuvant chemotherapy according to BRCA1 mRNA levels has been conducted in 88 completely resected stage II-III NSCLC patients. Patients with higher BRCA1 levels were treated with docetaxel, while cisplatin-based chemotherapy was reserved to those with lower BRCA1 levels. In the preliminary interim analysis, event free survival was similar in both groups [104]. Recently, a series of genes involved in DNA repair pathways and in metastasis formation were evaluated in 126 chemo-naive stage I- III A NSCLC patients who have received only surgical resection and were analyzed by RT-QPCR. For 77 patients with low levels of BRCA1, event-free survival has not been reached, while it was 22 months for those with high levels (P=0.04). For 83 patients with low levels of BRCA1, median survival has not been reached, while it was 29 months for those with high levels (P=0.04). The Cox proportional hazards model selected pathological stage IIIA and BRCA1 expression as independent prognostic factors for survival. The HR was 7.91 (95%CI, 2.27-27.54; P=0.001) for stage IIIA and 1.98 (95%CI, 1.11-6; P=0.02) for BRCA1 expression. In an independent validation cohort of 58 stage IB-IIB NSCLC patients, BRCA1 was also confirmed as the only independent prognostic markers [104]. BRCA1 mRNA expression may offer a supplementary information to tailored adjuvant chemotherapy for early NSCLC patients, but further analysis are necessary to clarify the role of this biomarker. 

### Gene Expression Profiles

Gene-expression profiling has recently been developed in several studies to indentify clusters of genes whose concomitant expression pattern may characterize prognosis, stratify risk and guide treatment decision for NSCLC patients [105-111].

Initially, genes have been identified as responsible for the aggressiveness of squamous cell carcinoma of the lung in a small group of stage I squamous cell carcinoma (SCCs), and also in other studies in which patients were unstratified for histopathologic subtype of lung cancer [105-107].

Gene expression profile has been analyzed in 51 completely resected stage I-III lung SCCs. Gene sets have been compared among individuals who remained disease-free for a minimum of 36 months with those from individuals whose disease recurred within 18 months of complete resection.

Using Cox proportional modelling, a 71-gene signature has been reported which was capable to predict recurrence and a set of 79-gene signature predictive for cancer-related death.

By joining these two signatures, a 111-gene signature was identified which was able to predict outcome in an independent test set of 58 SCCs with a 72% accuracy; this signature also predicted differences in survival [log-rank P= 0.0008; HR 3.8; 95% CI, 1.6–8.7]. This set of genes included genes with biological relevance to disease recurrence, such as cell growth and movement, cell communication and cell signalling [108]. In a similar clinical trial, it has been identified a 54-gene signature set in early stage adenocarcinoma lung cancer capable of predicting risk of recurrence in two independent validation cohorts. This signature predicted outcome independently of tumor stage and lymph-node [109].

Recently, it has been conducted a meta-analysis of datasets from seven different microarray studies on NSCLC for differentially expressed genes related to survival time (under 2 y and over 5 y). A gene signature consisting of 64 genes has been identified. Using this set of genes, it has been demonstrated a significantly worse OS for high-risk patients as compared to low-risk patients. These 64 genes includes eleven genes which are related to cancer metastasis and eight genes which are involved in apoptosis. The resulting signature is useful in predicting survival of stage I NSCLC and might be useful in making treatment decisions [110].

A total of 198 tumor specimes from three different cohorts of patients with early-stage NSCLC has been analayzed and a gene expression profile named the lung metagene model has been identified. These gene profile may predict a risk of disease recurrence with an accuracy of 93% in a cohort of 91 patients. This model was superior to a predictive model generated with the same methods but which includes only clinical characteristics (age, sex, stage, histology and smoking). When this model has been applied to the two validation cohorts, the predictive accuracy was superior to 70%. The lung metagene model also permits to identify a subgroup of stage IA NSCLC patients at high risk for recurrence. For these patients the survival rate at four years was less than 10%. However, the impact on response to chemotherapy in the high-risk group should be studied in a prospective randomized trial [111].

There is a lack of overlap in gene expression signatures in the several trials and a few or none genes were common to the different signatures. Moreover, at the moment none of the studies of NSCLC gene signatures so far published have been able to provide data to help the choice of chemotherapy in the adjuvant setting. 

## CONCLUSIONS

Improving outcomes for early-stage NSCLC is a major clinical research area, considering that a significant percentage of stage I patients develop recurrent disease within 5-years of complete curative lung resection. Conventional clinical prognostic markers fail to predict relapse accurately. The identification of predictive factors guiding the clinician’s choice, considering both the genetic profile of the patients and the biological characteristics of the disease could optimize the efficacy of current therapies in the adjuvant setting.

The discovery of genes that influence drugs activity and the toxicity related to treatment seems an interesting way to tailor individual therapy. The data summarized in this review are encouraging and indicate a progress towards the era of customized therapy. However, these data are not sufficient to support the routine use of pharmacogenetic markers in the clinical practice. In fact, several molecular markers have been tested by using using tissue samples from retrospective cohort studies; for these reason, further research is needed to identify valid and standardized laboratory methodologies for molecular assays and future prospective clinical trials using pharmacogenetic markers are required in the adjuvant setting.
